# Musculoskeletal disorders related to dental hygienist profession

**DOI:** 10.1111/idh.12596

**Published:** 2022-06-09

**Authors:** Matteo Saccucci, Giulia Zumbo, Paola Mercuri, Nicola Pranno, Selene Sotero, Francesca Zara, Iole Vozza

**Affiliations:** ^1^ Department of Oral and Maxillo‐Facial Sciences Sapienza University of Rome Rome Italy

**Keywords:** dental hygienist, MSDs, musculoskeletal disorders, professional disease

## Abstract

**Objectives:**

Musculoskeletal disorders (MSDs) are occupational illnesses concerned with different classes of professionals; dental hygienists are among those. The aim of this trial is to evaluate MSDs prevalence and significance of the symptoms in a sample of dental hygienists.

**Materials and Methods:**

A 20‐question questionnaire was administered to a sample of dental hygienists, via social networks. The variables taken into consideration were personal data, hours of sport, working habits, years of professional activity, working hours and number of patients per week, presence or absence of pain.

**Statistical Analysis:**

Data were evaluated using standard statistical analysis software, and an Excel database was created. Descriptive statistics were calculated for each variable. Group comparison was assessed by the chi‐square test of homogeneity and Fisher's exact test. (*p*‐value <0.05 as significant).

**Results:**

468 questionnaires were examined: 396 females (85%) and 72 males (15%). The prevailing age was between 25 and 35. Among them, 91% referred to be suffering or have suffered MSDs. The most relevant affected muscular areas are neck (30.6%), shoulder (25.0%) and lumbosacral region (23.3%); the remaining 21.1% is divided among the other regions. Association and statistical analysis among the different variables showed how presence of MSDs negatively influences absenteeism and work performance; further research regarding ergonomics, type of seat, stretching and workout prevention would be important to strengthen the results collected.

**Conclusions:**

Musculoskeletal disorders diffusion among dental hygienists is particularly high due to lack of information; the majority of interviewees showed very little awareness of it; this led to a lack of effort in facing or possibly preventing these pathologies.

## 1 INTRODUCTION

Musculoskeletal disorders (MSDs) are occupational pathologies that, unfortunately, affect several classes of professionals. They represent a relevant topic due to several reasons, such as absenteeism from work, which should not be underestimated. The muscular tone imbalance, which is the key in MSDs, causes musculoskeletal tensions too, concerning not only the physical sphere but also the psychological one. Although aetiology of MSDs is multifactorial, exposure to occupational risk factors significantly contributes to the onset of these disturbances.^1^ Among the causing factor, we can mention loads handling, repetitive movements, incorrect or static postures, vibrations and intense work routines. It is believed that these biomechanical risk factors, combined with psychosocial stress, contribute to the development and progression of MSDs.^2^, ^3^

Usually, the most affected muscular areas are neck, back and upper limbs, although in some rare cases lower limbs might be interested too. Symptoms of MSDs include discomfort, aching, numbness, tingling, burning, stiffness and fatigue. Signs of MSDs include decreased range of motion and grip strength, loss of normal sensation, movement and coordination.^4^

Pain from MSDs can occur in the neck, shoulder, arm, wrist, hands, upper and lower back, hips, knees and feet. Back pain in particular has been found to be a major health problem for dental professionals.^5^ Carpal tunnel syndrome (CTS) is one of the most common MSDs in dental hygienists causing numbness, tingling and pain in hand and wrist.^6^

According to the data from ‘The Work Foundation 2009, European Agency for Safety and Health at Work 2009’, it seems that the younger population is affected more, when compared to the ones that have a consolidated work experience instead.

Musculoskeletal disorders occur more often in those professions characterized by tasks that involve exposure to physical risk factors, as it was observed in some healthcare occupations.^1^ Among these last, dentists and dental hygienists seem to be more at risk than others.^4^, ^7^ A recent research supports this observation, with the prevalence of MSD in the dental profession at rates between 64% and 93%.^2^

Working as dental hygienists requires the adoption of fixed postures and use of repetitive and precise movements of fingers and hands. It is believed that these biomechanical risk factors, combined with psychosocial stress, contribute to the onset and progression of MSDs in dental hygienist professionals.^2^, ^3^

A 2001 study conducted in the United States found that 75.1% of 177 dental hygienists in the US army dental offices reported having hand problems, with 56.5% experiencing probable CTS symptoms.^6^ Compared with dentists, dental hygienists are at higher risk of developing an MSD in the upper extremities and lower back due to scaling and root planing over long hours.^8^ Additionally, dental hygienists have a higher prevalence of MSDs in the wrists and hands compared with dentists.^9^, ^10^

Other specific MSDs affecting dental hygienists are tension neck syndrome, thoracic outlet compression syndrome, pronator syndrome, tendonitis, lateral epicondylitis, trigger thumb, De Quervain's tenosynovitis, carpometacarpal osteoarthritis and vibration‐induced neuropathy.^2^ All of the above, leads to a loss of working time, a diminished working performance, necessity of medical investigation and possible earlier retirement.^11^, ^12^ Neck pain has specifically been identified as a common complaint of dental hygienists, with the 12‐month prevalence reported at between 54% and 69%.^13^, ^14^ Dental hygienists suffering from neck pain are more likely to have time off work or are considering reducing their working hours,^15^ affecting productivity and career longevity.

Considering this, the aim of this study was to examine the lifetime prevalence of MSDs and significance of symptoms in dental hygienists.

## 2 MATERIALS AND METHODS

### 2.1 Study design and sample

A cross‐sectional study was conducted in the form of an online questionnaire using the platform Google Forms. The survey was designed by a statistician and one of the authors and was evaluated in a previous pilot study used as thesis dissertation.^16^ There were 20 multiple‐choice questions: all of them were single answer, except the one regarding the body area affected by MSDs, where participants could choose more than one answer. The study population included all practising dental hygienists registered as active member at the Board of Dental Hygienists (AIDI) in northern, central and southern Italy. Students, non‐registered dental hygienists and respondents who did not answer any questions were excluded from this survey. The survey was administered in the period between March 2020 and August 2020 to a wide population of dental hygienists present on 3 chosen groups of the social network platform Facebook, and there was an equal distribution of dental hygienists who were invited across the aforementioned three regions.

Access to these groups was regulated so that only dental hygienists could have access to it. This platform was chosen for its easy accessibility and quick feedback. Questionnaire responses from a sample of 1000 hygienists were randomly selected using a data selection algorithm from the SPSS program (version 20.0 Statistical Package for the Social Sciences, IBM Corporation).

In order to improve the probability of finding a statistically significant result, power calculation was based on the percentage of interviewees which reported suffering or have suffered of MSDs evaluated in a previous pilot study used as thesis dissertation.^16^

The inclusion criteria for this study were being dental hygienist professional with at least 1 year of working experience and having properly filled the questionnaire (answered to more than 90% of the questions); both sexes were included.

The exclusion criteria were instead being non‐practising dental hygienist or being dental hygienists with less than 1 year of working experience; not having properly filled the questionnaire (answered less than 90% of the questions). Finally, three attempts were made to remind participants to complete the survey.

### 2.2 Questionnaire

The questionnaire (Appendix S1) was filled out anonymously and, in the participation and completion of it, the consent in the study was given.

The following information were asked to the participants:

- Age and sex.
- Training hours per week.
- Working habits: type of instruments used, type of chair, stretching habits, change of posture during worktime, use of loupes.
- Years of training.
- Working hours per week.
- Number of patients per week.
- Type of pain (when present): acute or chronic. The definition of acute and chronic pain was defined according to IASP (International Association for the Study of Pain ‐ 1986).
- Type of MSD reported (being able to choose among the six more common areas affected by MSDs).
- Absence from work due to MSDs.
- Need for medical care due to MSDs.

### 2.3 Statistical analysis

Data were evaluated using standard statistical analysis software (version 20.0, Statistical Package for the Social Sciences, IBM Corporation). A database was created using Excel (Microsoft). Descriptive statistics including mean ± SD values and percentage were calculated for each variable. Demographic characteristics were used to implement a stratification protocol in order to select a representative sample of the dental hygienist population.

The relationship between the following categorial variables was explored: presence of MSDs and number of working hours; presence of MSDs and practice of sport activity; type of MSDs and age; type of pain and seek of the help of a specialist; presence of MSDs and days of leave from work; type of pain and days of leave from work; presence of MSDs and type of working seat; presence of MSDs and use of magnification systems; presence of MSDs and practice of stretching.

Group comparison was assessed by the chi‐square test of homogeneity and Fisher's exact test. A *p*‐value of <0.05 was considered as statistically significant.

### 2.4 Ethical approval

The protocol was in accordance with the 1975 Declaration of Helsinki on medical protocols and ethics and its later amendments. No ethical committee approval was sought to start this study since this was not required by national legislation or any ordinance of the local inspection authority. In any case, the study was approved by the Institutional Local Review Board of Sapienza University of Rome in Latina (Protocol n. 01/2020).

## 3 RESULTS

Among the 1000 dental hygienist randomly chosen, 330 did not send the questionnaire back, while the remaining 670 did send it back, with 468 having filled it properly (answered more than 90% of the questions).

The remaining 202 did not respond correctly to the questionnaire (have answered less than 90% of the questions) and were therefore excluded from the sample.

The population was composed by 396 females and 72 males, with an age range between 25 and 65 years.

Among the interviewees, 91% referred to be suffering or have suffered Musculoskeletal Disorders (MSDs), while the remaining 9% had never suffered from MSDs. Gender imbalance was evident: women suffering from MSDs were 91.18% (362) over 396 filling the questionnaire; among the males, instead, 84.72% (61) were the affected subjects, over the 72 examined.

Further data have been deduced: 46% practised from 1 to 3 h of sport weekly, compared with the 40% that did not at all; only 14%, practised more than 3 h of sports weekly, instead. Moreover, men practised sport activity with more intense routines, 8% of them more than three times per week, while among the women sample, 6% practised more than three times per week.

Among the subjects reporting to be suffering of MSDs, 92.1% did not train at all during the week, 89.7% trained 1–3 h per week and 86.5% trained more than 3 hours per week. Although there was no significant difference between the number of hours of training and the presence of MSDs (*p*‐value = 0.371).

When it comes to the years of work experience, 41% of the interviewees declared to be working from 1 to 5 years, and 40% from more than 10 years. In terms of working hours per week, 53% of the interviewees worked full time (more than 30 h), 41% worked between 16 and 30 h and just 6% less than 15 h.

The most affected muscular areas were neck (30.6%), shoulder (25.0%) and lumbosacral region (23.3%); the remaining 21.1% of MSDs reported was divided among the other affected regions (elbow, hand and wrist, twitching finger, non‐specified others).

As far as the association between age and presence of MSDs is concerned, the results showed that the lumbosacral region is the most negatively influenced by age: 43.20% in the 51–65 age range, 16.50% in the 36–50 age range and 23.20% in the 25–35 age range (Table 1). While for the other affected regions, the presence of MSDs was similarly distributed in the different age ranges (Table 1).

**TABLE 1:** Distribution of areas affected by MSDs according to three different age ranges: 25–35 years old, 36–50 years old, 51–65 years old

Areas affected by MSDs	Age			Total
	25–35	36–50	51–65	
Lumbosacral region				
Count	69	21	19	109
% within areas affected by MSDs	63.3%	19.3%	17.4%	100.0%
% within age	23.2%	16.5%	43.2%	23.3%
Shoulder				
Count	80	27	10	117
% within areas affected by MSDs	68.4%	23.1%	8.5%	100.0%
% within age	26.9%	21.3%	22.7%	25.0%
Neck				
Count	92	45	6	143
% within areas affected by MSDs	64.3%	31.5%	4.2%	100.0%
% within age	31.0%	35.4%	13.6%	30.6%
Hand and wrist				
Count	22	15	5	42
% within areas affected by MSDs	52.4%	35.7%	11.9%	100.0%
% within age	7.4%	11.8%	11.4%	9.0%
Elbow				
Count	3	7	0	10
% within areas affected by MSDs	30.0%	70.0%	0.0%	100.0%
% within age	1.0%	5.5%	0.0%	2.1%
Twitching finger				
Count	2	0	2	4
% within areas affected by MSDs	50.0%	0.0%	50.0%	100.0%
% within age	0.7%	0.0%	4.5%	0.9%
Non‐specified				
Count	29	12	2	43
% within areas affected by MSDs	67.4%	27.9%	4.7%	100.0%
% within age	9.8%	9.4%	4.5%	9.2%
Total				
Count	297	127	44	468
% within areas affected by MSDs	63.5%	27.1%	9.4%	100.0%
% within age	100.0%	100.0%	100.0%	100.0%

Among the subjects reporting to be suffering from MSDs, just 18.1% needed to take days off from work; among these ones, 53.2% suffered from chronic pain and 43.8% from acute. Statistical analysis showed no significant difference between these two (*p* = 0.615). Therefore, there is no direct association between the type of pain and absence from work (Table 2).

**TABLE 2:** Patients with acute/chronic pain and absent from work

Absence from work or need for medical care	Chronic pain	Acute pain	Total
No			
Count	172	177	349
% within absence from work or need for medical care	49.3%	50.7%	100.0%
% within the definition of acute and chronic pain	80.8%	83.1%	81.9%
Yes			
Count	41	36	77
% within absence from work or need for medical care	53.2%	46.8%	100.0%
% within the definition of acute and chronic pain	19.2%	16.9%	18.1%
Total			
Count	213	213	426
% within absence from work or need for medical care	50.0%	50.0%	100.0%
% within the definition of acute and chronic pain was	100.0%	100.0%	100.0%

Despite MSDs are disabling, when present, the professional tries to reduce at the minimum the days of absence from work.

Although the percentage of absenteeism rises in the ones affected by MSDs, compared with the ones not affected (18.0% versus 2.2%), being significant (*p* = 0.006) Table 3.

**TABLE 3:** Patients affected by MSDs and absent from work

Presence of MSD	Absence from work or need for medical care		Total
	No	Yes	
No			
Count	45	1	46
% within presence of MSD	97.8%	2.2%	100.0%
% within absence from work or need for medical care	11.5%	1.3%	9.8%
Yes			
Count	346	76	422
% within presence_of_MSD	82.0%	18.0%	100.0%
% within absence from work or need for medical care	88.5%	98.7%	90.2%
Total			
Count	391	77	468
% within presence of MSD	83.5%	16.5%	100.0%
% within absence from work or need for medical care	100.0%	100.0%	100.0%

Furthermore, among the subjects taking days of leave, 46.8% suffered from lumbosacral and 24.7% from neck‐related disturbs.

In reporting the pain as chronic or acute, the difference was minimal and it was quite similarly distributed for both sexes, being chronic for 44.4% of males and 51.0% of females (Table 4); in spite of that, the difference was not significant (*p* = 0.339).

**TABLE 4:** Male and female with acute/chronic pain

The definition of acute and chronic pain	Gender		Total
	Male	Female	
Chronic pain			
Count	28	185	213
Expected count	31.5	181.5	213.0
% within the definition of acute and chronic pain	13.1%	86.9%	100.0%
% within gender	44.4%	51.0%	50.0%
Acute pain			
Count	35	178	213
Expected count	31.5	181.5	213.0
% within the definition of acute and chronic pain	16.4%	83.6%	100.0%
% within Sex	55.6%	49.0%	50.0%
Total			
Count	63	363	426
Expected count	63.0	363.0	426.0
% within the definition of acute and chronic pain	14.8%	85.2%	100.0%
% within sex	100.0%	100.0%	100.0%

Among those defining their pain as chronic, 54.2% asked for a medical treatment, while 45.8% of those defining their pain as acute, sought the help of a professional this difference was significant (*p* = 0.029).

The results related to the type of seat used showed that 84.1% of the interviewees use a chair with seatback, 5.3% use a chair with seatback and armrests, 9.0% use the saddle chair and just 0.9% the ball.

As far as the association between type of chair and suffering from MSDs is concerned, there was not significant difference among them (*p* = 0.682). The percentage of the ones suffering from MSDs was respectively for each type of seat: 90.4% of the ones using a chair with seatback, 92.0% among the ones using the chair with seatback and armrests, 85.7% among the ones using the saddle chair and 100% among the ones using the ball.

Approximately, 40% of the participants reported using loupes. However, the occurrence of MSDs was not significantly different between those who routinely wear loupes (92.6%) compared with those who do not (88.5%) (*p* = 0.148).

When analysing the different MSDs‐affected areas, 60.6% of those with MSDs in the lumbosacral area did not use loupes, as well as the 53.8% and 61.5% affected in the shoulder and neck areas, respectively. In spite of that, also in this case the association was not significant (*p* = 0.723) compared between those wearing loupes versus those without loupes.

Stretching during working hours is not so common yet: just 25.6% of the interviewees practised specific exercises, and the majority were women (79%). Among the ones engaged in doing stretching exercises, 86% suffered from MSDs. The percentage of subjects suffering from MSDs and not practising stretching (91.4%) was slightly lower, but still not enough to prove this association to be significant (*p* = 0.135). Moreover, 71% of the interviewees declared to have the habit of taking a break during working hours, but in 60% of these cases the pause is not dedicated to stretching practice.

## 4 DISCUSSION

Association and statistical analysis among the different variables showed how presence of MSDs negatively influences absenteeism and work performance; among the variables analysed, the relation with exercise, stretching and sport proven to be important in reducing the presence of MSDs.

Compared with other studies present in the literature,^5^, ^6^, ^17^, ^18^ one of the most significant data of this trial is the one related to the areas of pain manifestations. This, in fact, was mainly concentrated on neck, shoulder and lumbosacral areas. The significance of these data comes from the fact that in the majority of the studies carried out before, such as in the case of Hayes et al in 2009,^5^ a higher incidence in hand‐/wrist‐related problems was observed, compared to other susceptible areas.^17^, ^18^ Other systematic reviews carried out some years ago in Sweden and United States, put in evidence greater pain symptoms related to hand and wrist areas in the professional class of dental hygienists: percentages are around 64% and 69%.^6^, ^19^, ^20^, ^21^ Our results suggest how several muscular areas are simultaneously affected, suggesting a negative and invalidating impact in the quality of life and work of the professional figure examined. People aged between 51 and 65 are mainly affected, instead, by pain in the lumbosacral district; moreover, this kind of pain turns out to be very disabling, as it is often chronical and needs medical/physiotherapeutic treatment. These last precautions are considered to be effective in alleviating and sometimes resolving the pain symptoms, in accordance with the study of Nemes et al.^22^

Despite research demonstrated a decrease of MSDs when practising sports,^23^ the mechanisms of these gained benefits in the muscular component are still quite unclear.^19^, ^24^ According to our study, although there was no significant difference between the number of hours of training and the presence of MSDs, slightly lower percentages of MSDs were reported from the ones practising more activity. It is not possible to affirm that sport can reduce the incidence of MSDs, but further investigations regarding its benefits and use as a preventive instrument to avoid the onset of MSDs is needed.^23^

Although other interesting results stand out from our study and are backed up from several others in literature,^25^, ^26^, ^27^, ^28^ regarding the association between stretching practice and a lower rate of MSDs. Even if this practice is not so common yet among the population taken into consideration lower rates of MSDs were reported from these professionals, compared to the ones not practising it. In fact, as affirmed by Rodrigues et al. (2014), strength exercises with intensity of 70–85% of Repetition Maximum performed in the workplace, three times a week for 20 min are able to reduce musculoskeletal pain in shoulders, wrists, cervical, thoracic and lumbar spine.^25^ Furthermore, this kind of approaches suggest that regular movement as important in reducing the negative impact of dental work, particularly of static postures. This includes regular movement and changing postures over the workday, as well as integrating exercise, stretching yoga and relaxation exercises. Exercise and stretching also make sense from a biomechanical standpoint, and further intervention appears to be an important current research need to support and confirm the associations with lower MSD rates found in the current and other studies.^26^, ^27^, ^28^

Another preventive strategy can be the one implementing the use of tools aimed at helping ergonomics in the work field^29^; that makes the professional more aware of the difference in the position assumed during working hours and the one that they actually should have. In fact, there are a lot of professionals suffering from this condition, not being fully aware of their incorrect posture, as explained in the study of Partido et al.^30^ Regarding this, there are several types of seats whose contribution regarding this aspect might be interesting to evaluate, even though in our sample few professionals use them. Although the differences were not significant, there is a type of ergonomic seat, the saddle chair, that would suggest—by the lower count of MSDs reported—how the ergonomics of the seat could influence the onset or at least the mitigation of MSDs, turning out to be more beneficial. Tools help to acquire better ergonomics are able to generate immediate benefits even in few months, so that further worsening can be avoided. Although to be more relevant and clearer, this analysis should have been carried out in further studies on wider and more various samples, as in this case the number of other ergonomics seat (e.g. gym ball or other alternative seats) was not enough to be taken into consideration. Among the precautions that can be useful in improving ergonomics, it is clear that the use of magnification systems makes not necessary to get closer to the patient, in this way, the operator does not need to move their head forward.^31^, ^32^, ^33^ In our sample, a smaller presence of MSDs and neck‐related symptoms were observed in the professionals wearing loupes as opposed to the ones that suffer from these symptoms and do not wearing them. The preventive use of these systems is, therefore, highly recommended, observing the proportional lower rate of MSDs in our sample; it is due to the fact that it takes to maintain an ergonomically correct posture with a subsequent decrease in the inflammation of neck and back muscles.^34^ Moreover, it can be affirmed that, the combined use of magnification and saddle chair, both considered to be superior compared with other ergonomic tools, brings to a decrease in MSDs incidence.^34^, ^35^

Reaching full awareness regarding ergonomics principles is one of the main goals to get to in the next few years, as it was affirmed in a study of Bedi et al and in some other studies in literature.^36^ Full awareness needs also to be reached educating students, that often report to encounter difficulties in managing the correct posture to assume.^12^, ^37^ It is clear that the future requirements have to be the ones of educating the professionals, through dedicated lectures, starting from their course degree journey as nearly the totality of the interviewees of this study turned out to be interested in the addition of lectures/seminars regarding ergonomics.

As far as the relevance of absenteeism is concerned, among the subjects reporting to be suffering from MSDs despite these are disabling, when present, as we said, our results show that the majority of professional tries not to take days of leave from work (82% versus 18%). Although when compared to the ones not affected, the percentage of absenteeism rises in the ones affected by MSDs being statistically significant and proving how MSDs can negatively affect the efficiency, the productive and economic aspects of the life of the professional. Therefore, the professional tends once more to disregard their own health in favour of the professional practice and work routine.^33^, ^36^

As far as the difference between acute and chronic pain is concerned, it was quite similarly distributed, both for the two types of pain and for the sexes. What stood out, was how the type of pain influenced the seeking or not of medical treatment, as the one suffering from chronic pain turned more to the help of a professional, if compared to the ones suffering from acute pain. Prevention before therapy is what we have to look for. An injured worker, in fact, has financial and social negative and significant costs. In this case, workers who suffer from MSDs can be considered like that.^1^ This can lead to an increase in the request of sick‐leave, or to a simple reduction in the number of working hours; in some exceptional cases the total leave of the profession is asked. An investigation on dental hygienists in United Sates reported that the ones showing pains and paresthesia were absent from work due to MSDs for an average of 5 weeks per year.^11^ Also, the days of leave asked for physiotherapist's or physiatrist's appointments resulted to have a negative impact. This, in fact, caused a reduction in salary and, on the other side, greater medical expenses. A more detailed study regarding the possible ways of reducing MSDs and their impact on the psychosocial aspect is necessary. A review carried out from Mulimani et al from 2018^6^ shows how, despite there are several studies in literature, none of them evaluates the ergonomics actions that could be undertaken in order to improve both organizational and psychosocial aspects. Furthermore, no significant study was identified proving the effectiveness of the actions taken on the long run. Therefore, this study needs to structure randomized studies with longer follow‐up, so that an effective result of the actions taken on MSDs can be evaluated.

In consideration of this, we should wait and hope for the project ‘Fit For Work’^38^ to bring out good results, given that its aim was to give globally priority to musculoskeletal diseases by 2020. These will be considered as priority among all the harmful non‐transmissible diseases, due to their significant impact on morbidity, co‐morbidity, loss of productivity, sanitary inequalities and social exclusion.

There are some limitations to this study. This is a survey and not an interventional study; a physical examination of the participants or tests regarding ergonomics or stretching exercises would be ideal for increased validity of future studies. The study could have been extended to a larger sample, using also other system of recruitment that would have included a wider demographic not using social media.

To fully achieve the effectiveness of the questionnaire created by our team, it will be necessary further investigation with another one, based on a larger number of questions and focused on specific statistics, with the aim to collect more information regarding the less represented classes, like the ones of the professionals aged over 35.

## 5 CONCLUSIONS

This study led us to the conclusions of how MSDs are largely common and how more awareness needs to be raised among professionals, as poor consciousness regarding this problem, brings to the lack or delay in adopting preventive measures. Lower rates of MSDs resulted when these preventive measures were adopted, but in order to obtain more significant results, they probably have to be applied following specific protocols. Nevertheless, further research will be necessary in order to obtain a proper epidemiological study of the problem, in order to identify the risk factors and their impact on occupation, heading towards focused actions.

The significant result regarding absenteeism in professionals affected by MSDs, proved how this problem can negatively affect different aspects of the life of the professionals and therefore how important is to prevent the onset of this work‐related disease.

Furthermore, it would be useful to properly investigate and scientifically prove the correlation between exercise and its role in creating benefits on MSD‐affected professionals. Raising awareness, implementing positive changes, including regularly stretching and standing up throughout the day, may help to minimize MSDs. Maintaining good physical health is essential in order to conduct properly a work routine without pain, contributing to an overall improved quality of life.

## 6 CLINICAL RELEVANCE

### 6.1 Scientific rationale for study

MDSs are one of the most common professional diseases and dental hygienists are among the workers more frequently affected by them. Multiple factors can influence the onset of these disturbs and the goal of this study was to evaluate which ones and how they can be prevented or reduced.

### 6.2 Principal findings

Nearly, the totality of the interviewed professionals suffers from MSDs and this negatively affects their performance at work and increases absenteeism.

### 6.3 Practical implications

Awareness should be raised about this topic and prevention measures and research regarding ergonomics, type of seat, stretching and workout prevention should be pursued.

## AUTHOR CONTRIBUTIONS

M.S. contributed to conceptualization and methodology; I.V. contributed to validation, supervision, project administration and resources; P.M. and S.S. contributed to formal analysis; S.S. contributed to investigation; N.P. and M.S. contributed to data curation; F.Z. and G.Z. contributed to writing—original draft preparation; G.Z. and M.S. contributed to writing—review and editing. All authors have read and agreed to the published version of the manuscript.

## CONFLICT OF INTEREST

The authors declare no conflict of interest.

## Supporting information


Appendix S1

## Data Availability

The data that support the findings of this study are available from the corresponding author upon reasonable request.
