# Supplementary material for: Musculoskeletal disorders related to dental hygienist profession
Source: Int J Dent Hyg. 2022 Jun 9;20(3):571–9. doi: 10.1111/idh.12596 (PMC9543870; doi:10.1111/idh.12596)
Supplement: Supplementary file 1 — Appendix S1 [file IDH-20-571-s001.pdf]

Questionnaire of Musculoskeletal Disorders related to Dental Hygienist Profession

1. **Sex**
   - ☐ Male
   - ☐ Female
2. **Age**
   - ☐ 25 - 35
   - ☐ 36 - 50
   - ☐ 51 - 65
3. **Years of professional experience**
   - ☐ 1 to 5 years
   - ☐ 6 to 10 years
   - ☐ More than 10 years
4. **Average number of patients per week di pazienti**
   - ☐ Less than 15
   - ☐ 16 to 35
   - ☐ More than 35
5. **Number of working hours per week**
   - ☐ Less than 15
   - ☐ 16 to 30
   - ☐ More than 30
6. **Hours of exercise/sport per week**
   - ☐ None
   - ☐ 1-3 hours
   - ☐ More than 3 hours
7. **Do you suffer, or have you ever suffered, from Musculoskeletal Disorders?**
   - ☐ Yes
   - ☐ No

8. **If yes, which one among the following?**

- Neck
- Shoulders or shoulder blades
- Lumbosacral region
- Hand-Wrist (Carpal tunnel syndrome)
- Other: ________

9. **You define your pain as:**

- Chronic
- Acute

10. **Did you have to take work leave due to Musculoskeletal Disorders?**

- Yes
- No

11. **Did you seek medical help and treatment due to Musculoskeletal Disorders?**

- Yes
- No

12. **In which procedure do you feel more tired?**

- Mechanical strumentation
- Manual strumentation
- Other:_____

13. **Do you prefer to use instruments with handle:**

- Wide diameter and lightweight
- Small diameter and heavier
- Other: _____

14. **Do you use magnification systems?**

- Yes
- No

15. **Which type of seat do you use?**

- Saddle chair
- Chair with seatback
- Chair with seatback and armrests
- Other: _____

16. **Do you usually practice stretching exercises?**
- ☐ Yes
- ☐ No

17. **Do you often change working position?**
- ☐ Yes
- ☐ No

18. **Do you takae breaks between patients' sessions?**
- ☐ Yes
- ☐ No
- ☐ Sometimes

19. **Do you feel the need to get closer to the patient in order to have a better visual?**
- ☐ Yes
- ☐ No

20. **Would you agree if a seminar regarding ergonomics education focused on posture, would be inserted into the Degree course curriculum?**
- ☐ Yes
- ☐ No
